# A revision of the genus *Hemitrochostoma* (Hemiptera, Heteroptera, Plataspidae)

**DOI:** 10.3897/zookeys.495.8861

**Published:** 2015-04-08

**Authors:** Dávid Rédei, Zdeněk Jindra

**Affiliations:** 1 Institute of Entomology, College of Life Sciences, Nankai University, Weijin Road 94, Tianjin, 300071, China; 2 Department of Zoology, Hungarian Natural History Museum, H-1088 Budapest, Baross u. 13, Hungary; 3 Department of Plant Protection, Faculty of Agrobiology, Food and Natural Resources, Czech University of Agriculture, CZ-165 21 Praha 6 – Suchdol, Czech Republic

**Keywords:** Taxonomy, Heteroptera, Plataspidae, *Hemitrochostoma*, Oriental Region

## Abstract

The genus *Hemitrochostoma* Bergroth, 1913 (Hemiptera, Heteroptera, Plataspidae) is redescribed and reviewed. Two species are recognized: the type species *Hemitrochostoma altilabris* Bergroth, 1913 (Malaysia: Sarawak), and *Hemitrochostoma rutabulum* **sp. n.** (Malaysia: Perak). The following new subjective synonymies are proposed: *Hemitrochostoma* Bergroth, 1913 = *Inflatilabrum* Tomokuni, 2012, **syn. n.**; *Hemitrochostoma altilabris* Bergroth, 1913 = *Inflatilabrum lambirense* Tomokuni, 2012, **syn. n.**

## Introduction

The genus *Hemitrochostoma* Bergroth, 1913 (Hemiptera, Heteroptera, Plataspidae) has remained monotypic so far; the single included species, *Hemitrochostoma altilabris* Bergroth, 1913 was described from Borneo, Malaysia. Except for a paper briefly discussing the peculiar morphology of *Hemitrochostoma altilabris* (Rédei & Bu, 2013) we could not trace any literature mentioning the genus or species. *Hemitrochostoma* and its included species are redescribed, an additional new species is described, and new genus and species-level synonymies are proposed in the present paper.

## Material and methods

External structures were examined using a stereoscopic microscope (Zeiss Discovery.V8). Drawings were made with the aid of a camera lucida. Genitalia were dissected after careful maceration in KOH, stained with Chlorazol Black E if necessary, and examined using stereoscopic (Zeiss Discovery V8) and optical (Olympus CX21) microscopes. Digital photographs were taken with a Nikon D90 camera equipped with an AF-S Micro Nikkor 60mm f/2.8G ED lens.

Measurements were taken with calibrated micrometer eyepiece. Since the apical portion of the mandibular plates are strongly curved upwards, the length of the head was measured to the apex of the anteclypeus in dorsal position of the frontoclypeus, and to the apex of the mandibular plates in the most exposed (anterodorsal) position of the head.

The two species of this genus are highly similar in their colour, integument, vestiture, and external morphology. Therefore in order to avoid repetitions a rather detailed description of the genus is provided; the species descriptions are kept short and focus on the diagnostic characters of the two species. Morphological terminology mostly follows Tsai et al. (2011).

Abbreviations for depositories: HNHM, Hungarian Natural History Museum, Budapest, Hungary; NSMT, National Museum of Nature and Science (formerly National Science Museum), Tokyo, Japan; ZJCP, Zdeněk Jindra’s private collection, currently deposited at the Department of Plant Protection, Czech University of Agriculture, Prague, Czech Republic.

## Taxonomy

### Genus Hemitrochostoma

Taxon classificationAnimaliaHemipteraPlataspidae

Bergroth, 1913

Hemitrochostoma Bergroth, 1913: 176. Type species by monotypy: *Hemitrochostoma altilabris* Bergroth, 1913.
Inflatilabrum Tomokuni, 2012: 40. Type species by original designation: *Inflatilabrum lambirense* Tomokuni, 2012. **New subjective synonym.**

#### References.

Rédei and Bu 2013: 272 (morphology).

#### Diagnosis.

**Figures 1–4.**
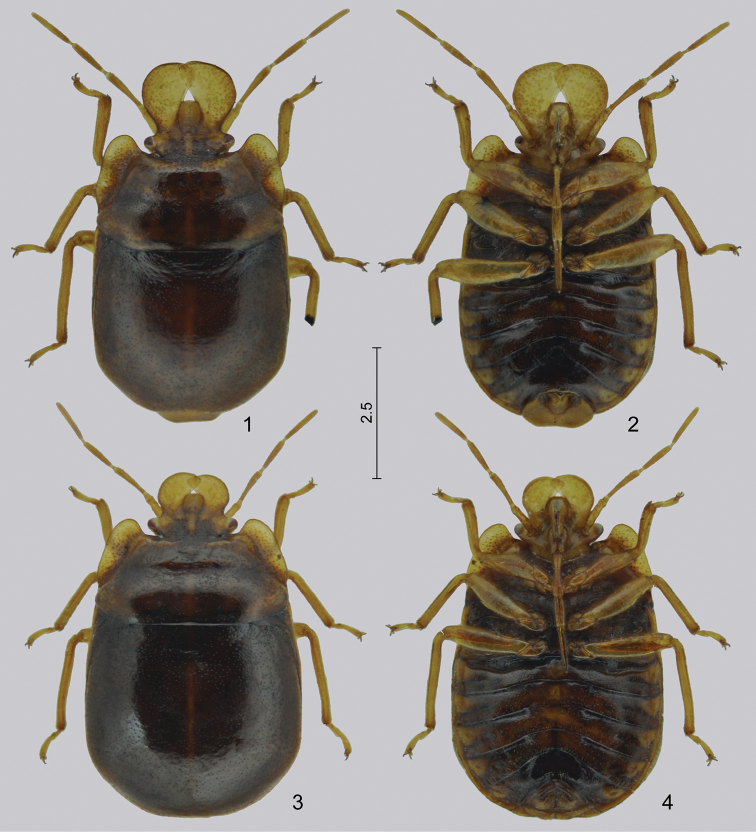
*Hemitrochostoma rutabulum* sp. n., male **1–2** and female **3–4. 1**, **3** dorsal view **2**, **4** ventral view. Scale in mm.

**Figures 5–8.**
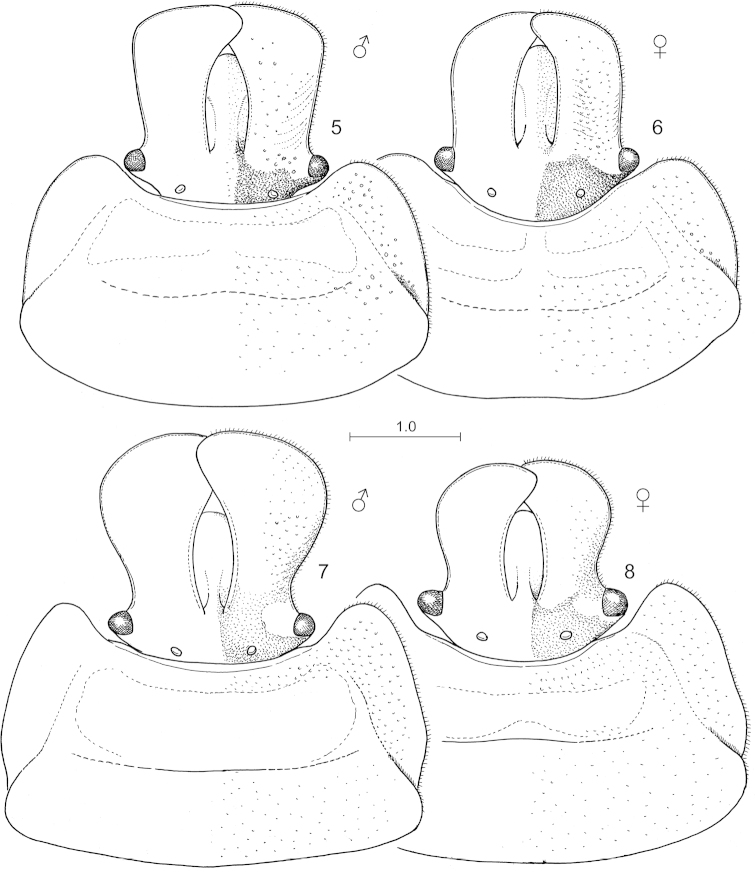
*Hemitrochostoma* species, head and pronotum in most exposed view. **5** *Hemitrochostoma altilabris* Bergroth, 1913, male **6** same, female **7** *Hemitrochostoma rutabulum* sp. n., male **8** same, female. Pilosity only shown along margin of head and pronotum. Scale in mm.

Recognized within Plataspidae based on the combination of the following characters: body of medium size (6–7 mm), relatively elongate, about 1.5–1.8 times as long as wide, moderately convex dorsally (Figs 1–4); mandibular plates broadly laminate, produced far anteriad of apex of anteclypeus and broadly overlapping in both sexes (Figs 5–8); labrum highly elevated, crest-like, semicircular in lateral view (Fig. 11).

**Figures 9–11.**
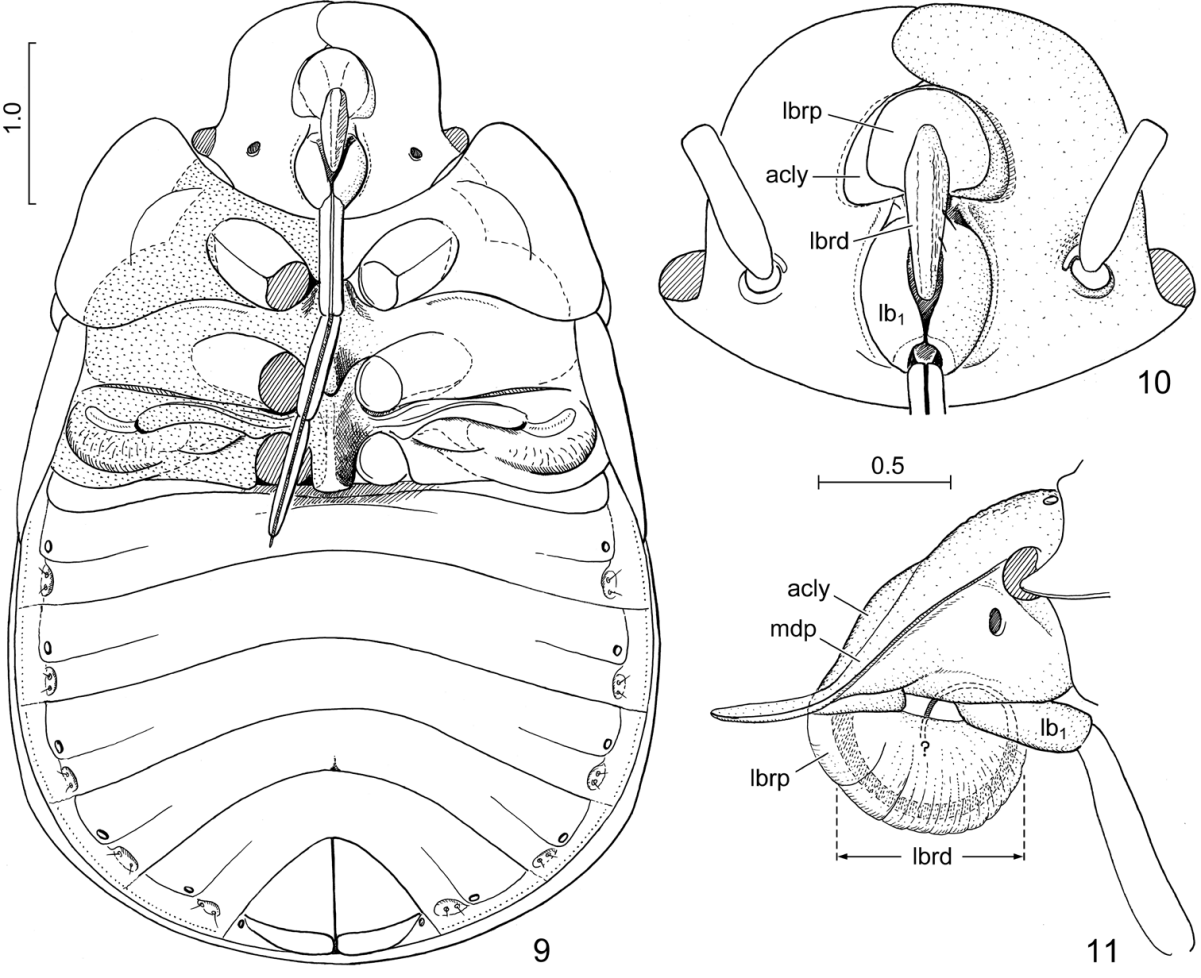
*Hemitrochostoma altilabris* Bergroth, 1913, female. **9** body in ventral view, antennae and legs removed, labium pulled to right, thoracic evaporatorium densely dotted **10** head, ventral view **11** same, lateral view. Lettering: acly = anteclypeus; lb_1_ = labial segment I; lbrd = distal portion of labrum; lbrp = proximal portion of labrum; mdp = mandibular plate. Scales in mm.

#### Redescription.

*Body* of medium size (total length about 6–7 mm), elongately oval, moderately convex dorsally, nearly flat ventrally (Figs 1–4). *Colour, integument and vestiture.* Dorsum brown with stramineous areas on head and pronotum; integument dull, smooth, with dense, weak, superficial, rather irregular punctation dorsally, with only insignificant, indistinct punctures ventrally; with scattered, inconspicuous, fine, short, erect setae dorsally and ventrally, antennae and legs with more conspicuous short, semierect pilosity.

*Head and cephalic appendages. Head* (Figs 5–8) 1.05–1.1 times as long as its width across eyes, about as long as or slightly longer than median length of pronotum, about half as broad across eyes as humeral width of pronotum, width across eyes 1.3–1.35 times (male, female) as broad as interocular distance; sexually dimorphic: males (Figs 5, 7) with more strongly broadened mandibular plates than females (Figs 6, 8); anteclypeus thick, with lateral margins arched, therefore anteclypeus distinctly broader at its middle than apically and basally, dorsally elevated above level of mandibular plates (Fig. 11); mandibular plates broadly, laminately produced, far surpassing apex of clypeus, overlapping medially anteriad of clypeus but separated from apex of clypeus by a distinct gap, lateral margin of mandibular plates sharp, reflexed, distinctly (*Hemitrochostoma altilabris* male, *Hemitrochostoma rutabulum* male, female) or insignificantly (*Hemitrochostoma altilabris* female) emarginate anteriad of eye, portion anteriad of apex of clypeus strongly turned upwards; antennal insertion situated closer to mesal margin of eye than to base of labium (Fig. 10); buccula short, restricted to basal half of ventral side of head; labrum subdivided into a broadly inflated proximal portion (Figs 10–11: lbrp) and a highly elevated, crestlike distal portion semicircular in lateral view (Figs 10–11: lbrd); compound eyes very small (male, female), distinctly protruding laterally; ocelli small, interocellar distance about 2 times as long as distance between ocellus and ipsilateral eye. *Antenna* simple, segment II subdivided into two secondary segments, segment I not reaching apex of head, thickened at its basal half, segment IIb very short, ring-like, segments IIb–IV distinctly flattened. *Labium* reaching or surpassing posterior margin of abdominal ventrite III; segment I (Figs 10–11: lb_1_) thick and short, not reaching base of head, diameter of segments II–IV much smaller, segment III distinctly longer than segment I, segments II and IV subequal in length, both distinctly longer than segment I.

*Thorax and thoracic appendages. Pronotum* (Figs 5–8) more than two times as broad as its median length, moderately declivous anteriorly; anterior collar narrow but distinct; lateral margin broadly, laminately explanate, more or less strongly produced anterolaterad reaching or surpassing anterior margin of eye, gradually narrowed posteriad, more or less emarginated anteriad of humeri in dorsal view; humeral angle rounded, obtuse, distinct; posterior margin broadly rounded, posterolateral angle obsolete. *Scutellum* wider than long, lateral margins abruptly broadened at their extreme base, then moderately broadening posteriad, forming a broadly rounded angle at two thirds of its length, then more strongly narrowing towards apex; basal tumescence weak, posteriorly delimited by a broad and very shallow transversal furrow; disk with a very indistinct median carina; basolateral angle not delimited by furrow; with a fine submarginal impression along almost entire length except at extreme base; posterior margin shallowly (female) or more deeply (male) excised above terminalia. *Thoracic pleura and sterna* (Fig. 9). Proepisternum simple, not tumescent; metapleuron with a well developed scent gland ostiole situated about halfway between base of hind coxa and dorsal margin of metapleurite, associated with a distinct, well developed but rather short, highly elevated, elongate, weakly curved peritreme; mesosternum forming a broad, obtuse, rather low median carina, posterior margin V-shaped and distinctly produced between mid coxae; metasternum relatively narrow, elevated and somewhat tumescent, but meso- and metasterna not forming a contiguous carina. Evaporatorium occupying most of thoracic pleuron and sternum except broad lateral explanate margin of propleuron, adjacent broad region of proepisternum, and pro-and mesothoracic supracoxal lobes. *Fore wing.* Exocorium and adjacent small, triangular basal portion of mesocorium exposed in rest. *Legs* short, femora thickened, tibiae with distinct, broad, deep dorsal furrow along their whole length.

*Pregenital abdomen* (Figs 9, 26) distinctly broader than long; connexival segments distinctly separated, intersegmental sutures running to lateral margin of abdomen; posterolateral angles of segments IV and V minutely, obtusely, protruding; dorsal laterotergites present in segments III–VI, rather broad; ventral laterotergites greatly fused with the respective sternites (these ventral plates of composite origin are termed ventrites), lateral portions of ventrites III–VII demarcated by a longitudinal furrow, spiracles III–VII situated close to this furrow mesally and about halfway between anterior and posterior margin of each segment; segments III–VII each with 2 pairs of longitudinally arranged trichobothria situated posteriad and somewhat mesad of the respective spiracles in an oval impression; median lengths of ventrites III–VI subequal, that of ventrite VII somewhat longer (female); posterior margin of ventrite VII of female deeply, subtriangularly emarginate (cf. Figs 26, 28, 32); tergite VIII (Fig. 27: t_8_) short, narrowly desclerotized along midline.

*External male genitalia*. Genital capsule (Figs 12–15) relatively small (more narrow than width of head), robust, simple, with dorsal sinus elongate. Paramere (Figs 16–17, 22–23) simple, relatively narrow, S-shaped, gradually tapering towards apex. Phallus described in detail for *Hemitrochostoma altilabris*.

**Figures 12–15.**
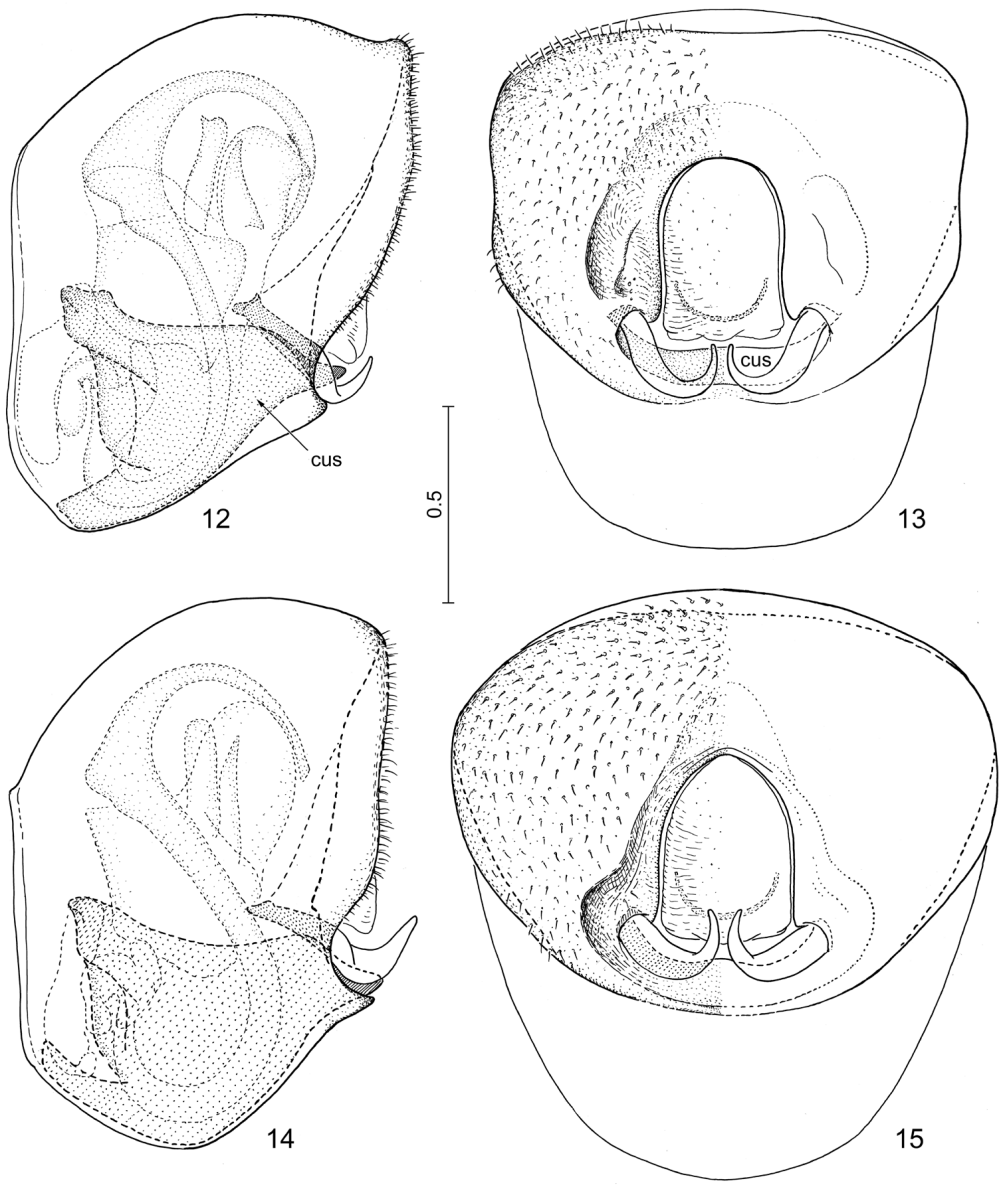
*Hemitrochostoma* spp., genital capsule with phallic organs and postgenital abdomen. **12** *Hemitrochostoma altilabris* Bergroth, 1913, posterior view **13** same, posterior view **14** *Hemitrochostoma rutabulum* sp. n., lateral view **15** same, posterior view. Lettering: cus = cuplike sclerite. Scale in mm.

**Figures 16–23.**
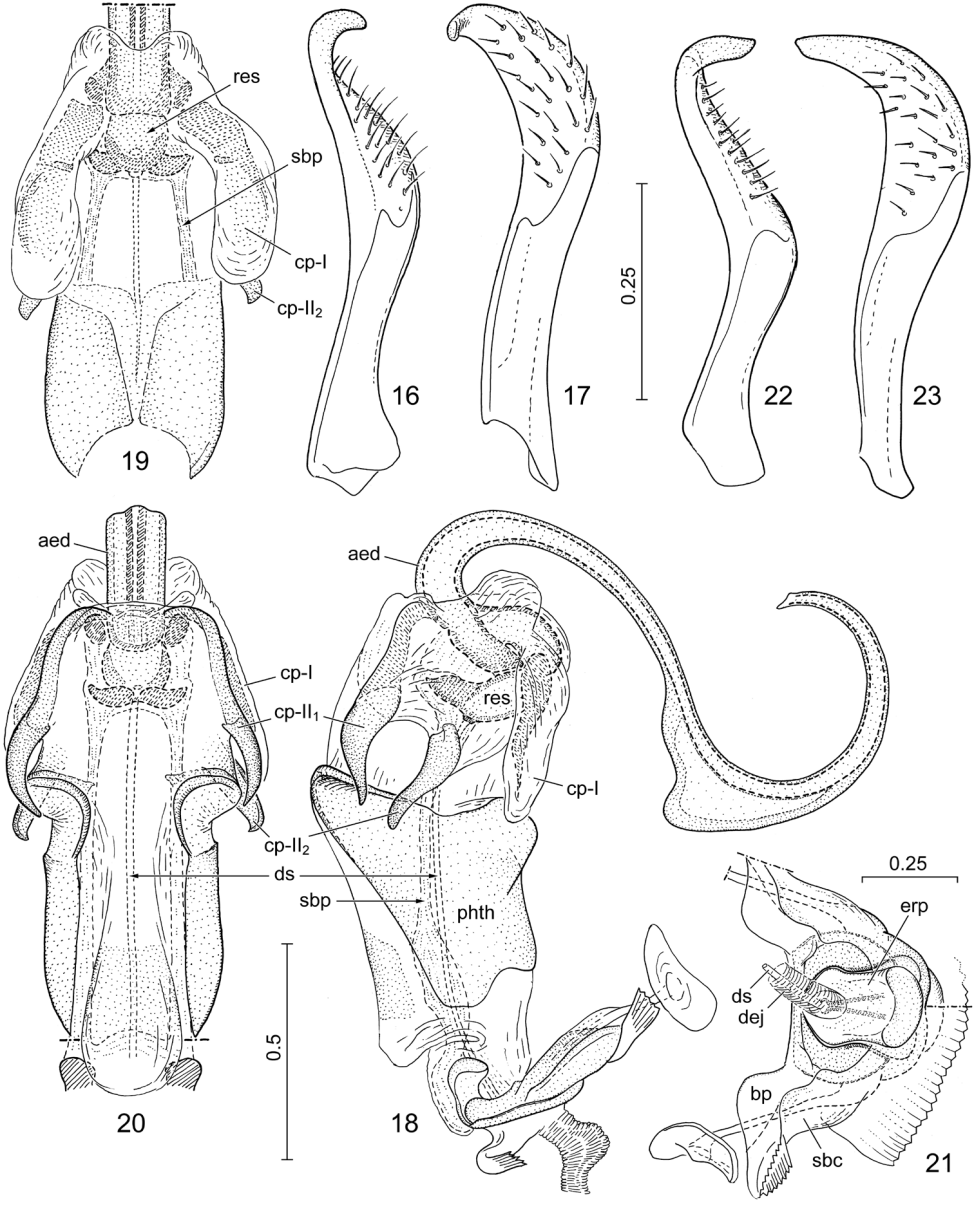
*Hemitrochostoma* spp., external male genitalia. **16–21** *Hemitrochostoma altilabris* Bergroth, 1913 **22–23** *Hemitrochostoma rutabulum* sp. n. **16–17**, **22–23** left paramere **18** phallus, lateral view **19** same, dorsal view, articulatory apparatus and most of aedeagus removed **20** same, ventral view **21** articulatory apparatus. Lettering: aed = aedeagus, bp = basal plates, cp-I, cp-II_1_, cp-II_2_ = conjunctival processes (see text), dej = ductus ejaculatorius, ds = ductus seminis, erp = erection fluid pump, res = endophallic reservoir, sbc = support bridge complex, sbp = support bridge prolongation. Scales in mm.

*External female genitalia* (Figs 28–31). Valvifers VIII subtriangular; laterotergites IX subhorizontal, mesal margin subtruncate; ectodermal genital tracts described in detail for *Hemitrochostoma altilabris*.

**Figures 24–27.**
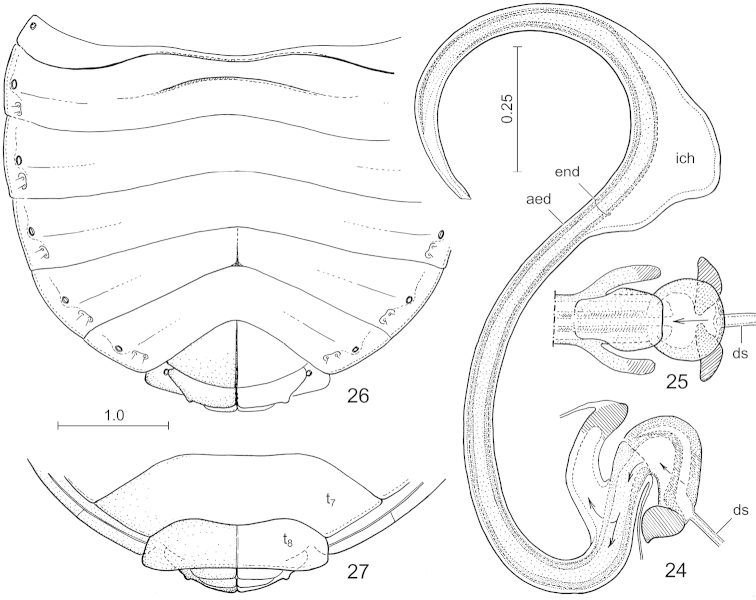
*Hemitrochostoma altilabris* Bergroth, 1913. **24** aedeagal complex, lateral view **25** proximal portion of the aedeagal complex, dorsal view **26** abdomen of female, ventral view **27** same, posterior portion, dorsal view. Lettering: aed = aedeagus, ds = ductus seminis, end = endophallic duct, ich = inner chamber, t_7_, t_8_ = tergites VII and VIII. Arrows in Figs 24–25 show the pathway of the sperm. Scales in mm.

**Figures 28–32.**
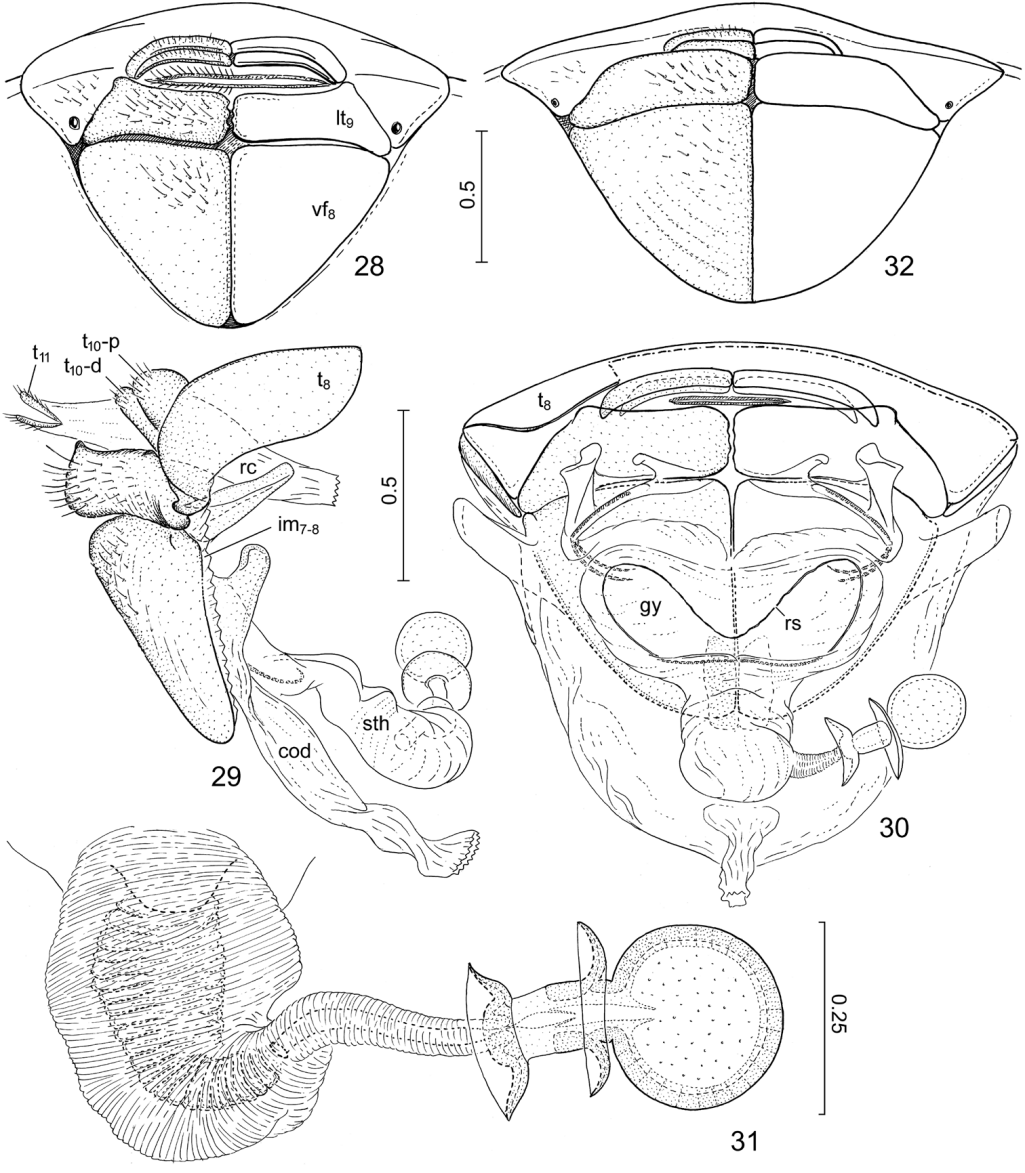
*Hemitrochostoma* species, external female genitalia. **28–31** *Hemitrochostoma altilabris* Bergroth, 1913 **32** *Hemitrochostoma rutabulum* sp. n. **28**, **32** terminalia, posteroventral view **29** individualized segments VIII–XI, lateral view **30** same, anterodorsal view **31** spermatheca. Lettering: cod = common oviduct, gy = gynatrium, im_7-8_ = intersegmental membrane between segments VII and VIII, lt_9_ = laterotergite IX, rc = rectum, rs = ring sclerite, sth = spermatheca, t_8_ = tergite VIII, t_10_-p, t_10_-d = proximal and distal portions of tergite X, respectively, t_11_ = tergite XI, vf_8_ = valvifer VIII. Scales in mm.

*Postgenital abdomen*. Male: proctiger elongate, concealing dorsal sinus of posterior aperture of genital capsule in rest, weekly trilobate apically (Figs 13, 15). Female: segment X transversally subdivided into two secondary segments (Fig. 29: t_10_-p, t_10_-d) and each of these secondary segments subdivided into a pair of contralateral sclerites along midline.

#### Distribution and diversity.

The genus previously contained only the type species occurring in northern Borneo. Another species is described in the present paper from Peninsular Malaysia (Perak).

**Figure 33.**
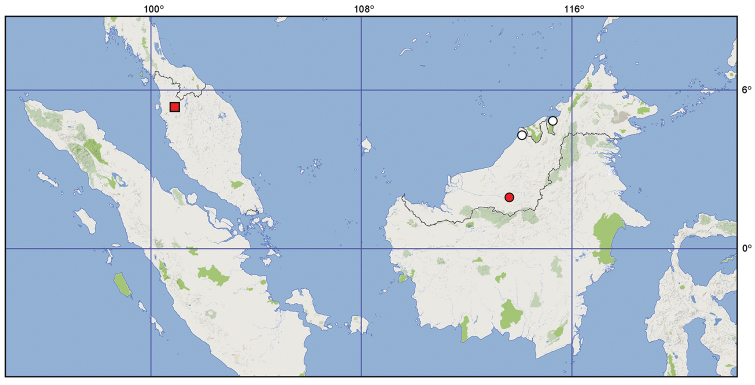
Distribution of *Hemitrochostoma* species. Circles: *Hemitrochostoma altilabris* Bergroth, 1913; square: *Hemitrochostoma rutabulum* sp. n. Red symbol represent record based on specimens examined by us, white symbols represent literature data.

### Hemitrochostoma altilabris

Taxon classificationAnimaliaHemipteraPlataspidae

Bergroth, 1913

Figs 5–6 , 9–11 , 12–13 , 16–21 , 24–27 , 28–31

Hemitrochostoma altilabris Bergroth, 1913: 177. Syntype(s) (male): [Malaysia:] Borneo, Sarawak, Trusan; depository unknown (see Discussion).
Inflatilabrum lambirense Tomokuni, 2012: 40. Holotype (male): Malaysia: Borneo, Sarawak, Lambir Hills National Park, Inoue Trail; NSMT. **New subjective synonym.**

#### References.

Rédei and Bu 2013: 272 (morphology).

#### Material examined.

**MALAYSIA. Borneo: Sarawak**, Kapit Distr., env. of Rumah Ugap, valley of Sut River, 3–9.iii.1994, leg. P. Bílek (3 males 3 females ZJCP, 1 male 1 female HNHM).

#### Diagnosis.

Differs from the only known congener, *Hemitrochostoma rutabulum* sp. n., by the less-dilated mandibular plates in both sexes and the less-produced anterior angles of the pronotum (not extending to anterior margin of eye in dorsal view) (Figs 5–6).

#### Redescription.

Male and female. Photographs of the habitus and diagnostic characters of both sexes were presented by Tomokuni (2012) under the name *Inflatilabrum lambirense*.

*Colour and integument.* Dorsum of body brown, following parts ochraceous: mandibular plates except their narrow lateral margin, a middorsal vitta between base of clypeus and base of head usually narrowing towards base of head; explanate lateral margin, a pair of transverse submedian fasciae between collar and calli, some suffused spots at posterior margin of calli, and humeral tubercles on pronotum; a pair of distinct or indistinct spots at basal tumescence and occasionally an indistinct median vitta on disk of scutellum; visible portion of costal margin of fore wing; lateral and posterior margins of scutellum broadly marmorated with ochraceous of various extent; legs ochraceous, antennae slightly darker, brownish ochraceous; venter of head brown, ventrally visible surfaces of mandibular plates and clypeus ochraceous, labium ochraceous, ventrally visible portions of thorax and abdomen brown, explanate lateral margin of propleuron ochraceous, abdominal ventrites somewhat lighter brown than pterothoracic pleura, lateral margin and an adjoining sublateral spot at anterior margin of each of ventrites III–VII ochraceous. Integument and vestiture as in the generic description.

*Structure. Head* (Figs 5–6) with lateral margin of mandibular plates weakly (male) or insignificantly (female) emarginate anteriad of eye, then more or less straight, slightly diverging anteriad (male) or subparallel (female), apically abruptly truncate (male, female). *Pronotum* (Figs 5–6) with anterolateral angles distinctly produced anteriad but not reaching anterior margin of eye (male, female). Other characteristic of the exoskeleton as in generic description. A detailed description, habitus photos and photos of diagnostic characters of both sexes were provided by Tomokuni (2012) under the name *Inflatilabrum lambirense*.

*External male genitalia.* Genital capsule (Figs 12–13) with a pair of weak lateral notches in posterior view, therefore lateral margins appearing weakly emarginate in most exposed view; cuplike sclerite (Fig. 12–13: cus) large, occupying most of ventral inner surface of genital capsule, visible as a transverse rim-like plate along ventral margin of posterior aperture of genital capsule in posterior view. Paramere as in Figs 16–17. Phallus (Figs 18–21, 24–25): support bridge complex (Fig. 21: sbc) and basal plates (Fig. 21: bp) strongly fused, without window-like interspace between them, each with a pair of robust, laterally directed arms dorsally; erection fluid pump (Fig. 21: erp) short; support bridge prolongation (Figs 18–19: sbp) forming a pair of rather thin bands running parallel with ductus seminis (Figs 18, 20: ds); phallotheca (Fig. 18: phth) dorsally and laterally sclerotized, ventrally membranous, sclerotized lateral wall produced into a pair of rounded lobes ventrodistally (cf. Figs 18, 20); conjunctiva voluminous, greatly membranous, provided with a pair of dorsolateral membranous, partially sclerotized processes (probably homologous with cp-I of Tsai et al. 2011) (Figs 18–20: cp-I) and a pair of lateral processes which are broadly membranous proximally but terminating in 2 strongly sclerotized, long, spine-like projections distally (Figs 18–20: cp-II_1_ and cp-II_2_); endophallic reservoir (Figs 18–19: res) small, with a pair of ventral protuberances associated with distal parts of support bridge prolongation, separated by basal portion of aedeagus s. str. by a deep, narrow incision; aedeagus s. str. (Figs 18, 20, 25: aed) greatly elongate, narrow, tubular, strongly curved in an S-shape in the sagittal plane, narrowly enclosing the tubular endophallic duct (Fig. 24: end) in most of its length except in the area of a strongly elevated, carina-like dorsal lump immediately distad of its middle which encloses an inner chamber (Fig. 24: ich); apex of aedeagus s. str. obliquely truncate.

*External female genitalia.* Posterolateral margins of valvifers VIII (Fig. 28: vf_8_) (adjacent with ventrite VII) nearly straight; laterotergites IX (Fig. 28: lt_9_) subhorizontal, relatively narrow; gynatrium (Fig. 30: gy) short, transverse, contralateral ring sclerites (Fig. 30: rs) fused into a single uninterrupted ring; spermatheca (Fig. 29: sth, Fig. 31): proximal portion of spermathecal duct broad, thick-walled, with a broad lumen, gradually narrowed in its middle portion, distally narrow, tubular; intermediate part of spermatheca short and thick, flexible zone occupying about its proximal half; proximal and distal flanges relatively small; distinct septum and fretum present; apical receptacle simple, globose.

*Measurements* (in mm) (N = 3 males / 3 females). Body length 6.2–6.5 / 6.0–6.4; median length of head to apex of clypeus 1.48–1.54 / 1.42–1.50, to apex of mandibular plates 1.68–1.80 / 1.70–1.75, greatest width across mandibular plates subapically 1.52–1.58 / 1.38–1.42, width across eyes 1.76–1.78 / 1.78–1.81, interocular distance 1.34–1.36 / 1.33–1.39, interocellar distance 0.66–0.67 / 0.64–0.71, oculo-ocellar distance 0.27–0.30 / 0.30–0.33; lengths of antennal segments I: IIa: IIb: III: IV as 0.59–0.68 / 0.58–0.64: 0.10–0.12 / 0.09–0.10: 0.96–1.01 / 0.93–0.96: 0.80–0.84 / 0.82–0.86: 0.92–0.95 / 0.86–0.96; median length of pronotum 1.62–1.66 / 1.60–1.72, humeral width 3.41–3.50 / 3.56–3.68; median length of scutellum 3.08–3.10 / 3.16–3.45, greatest width 3.58–3.66 / 3.92–4.00.

#### Immatures.

Description and photograph of the 5^th^-instar larva were presented by Tomokuni (2012) under the name *Inflatilabrum lambirense*.

#### Distribution.

The species is known only from a few localities, all in Sarawak, Borneo, Malaysia. — **MALAYSIA. Borneo: Sarawak:** env. of Rumah Ugap!, Trusan (Bergroth 1913), Lambir Hills National Park (Tomokuni 2002) (Fig. 33).

#### Bionomics.

The bionomics of *Hemitrochostoma altilabrum* (as *Inflatilabrum lambirense*) was discussed by Tomokuni (2012). The species feeds on sap of the trunks of *Dryobalanops aromatica* C.F.Gaertn. (Dipterocarpaceae) and another, unidentified tree species. Adults and larvae live in shelters under dead peeling bark of the lower regions of the trunk created by mutualistic ants (Formicidae: *Camponotus* spp.).

#### Discussion.

*Hemitrochostoma altilabris* was described based on an unspecified number of specimens (all males). The type depository was not indicated in the original description (Bergroth 1913). The type(s) could not be located in the Zoological Museum of the University of Helsinki (L. Huldén, pers. comm.) currently housing most of the types of E. Bergroth (Schuh and Slater 1995), and also was not found in other important depositories of Bergroth types: the Zoological Museum of the Humboldt University of Berlin (J. Deckert, pers. comm.), the Senckenberg Deutsches Entomologisches Institut in Müncheberg (S. Blank, pers. comm.), the HNHM, the Naturhistorisches Museum in Vienna, and the Natural History Museum in London (all of them visited by DR). Because the specimens examined by us were collected rather close to the type locality, and the males are in agreement with the description and illustrations of Bergroth (1913), they are readily identified as *Hemitrochostoma inflatilabrum*. Although the type material currently cannot be located, so far there is no doubt about the identity of this species and, therefore, designation of a neotype is not justified.

The detailed original description and illustrations of *Inflatilabrum lambirense* leave no doubt that this species is conspecific with *Hemitrochostoma altilabris*; therefore, the following new subjective synonymies are proposed: *Hemitrochostoma* Bergroth, 1913 = *Inflatilabrum* Tomokuni, 2012, syn. n.; *Hemitrochostoma altilabris* Bergroth, 1913 = *Inflatilabrum lambirense* Tomokuni, 2012, syn. n.

### Hemitrochostoma rutabulum sp. n.

Taxon classificationAnimaliaHemipteraPlataspidae

http://zoobank.org/6DFEDCDD-916B-45A6-B33D-24169E88FA6E

Figs 1–4 , 7–8 , 14–15 , 22–23 , 32

#### Type material.

**Holotype** (male): “MALAYSIA - Perak \ Banjaran Bintang \ Maxwel Hill (Taiping) \ 18. - 19.2.1997 \ Ivo Jeniš”. Mounted on card, tarsus and apical half of tibia of right hind leg lacking, genitalia preserved in plastic microvial filled with glycerol pinned with the specimen, deposited in ZJCP. Paratype: same label as for holotype; intact (1 female, ZJCP).

#### Diagnosis.

Easily distinguished from the only known congener, *Hemitrochostoma altilabris* by the much more strongly dilated mandibular plates, particularly in the male (Fig. 7) but also in the female (Fig. 8), and the more produced anterior angles of the pronotum (surpassing anterior margins of eyes in both sexes) (Figs 7, 8). The ground colour of the two examined specimens is considerably darker than in *Hemitrochostoma altilabris*, and the scutellum is not marmorated with ochraceous along its lateral and posterior margin.

#### Description.

Macropterous male and female.

*Colour and integument.* Dorsum of body brown, following parts ochraceous: mandibular plates, clypeus and a confluent middorsal vitta running to base of head; explanate lateral margin, a pair of transverse submedian fasciae between collar and calli, weak suffusion at posterior margin of calli and on humeral tubercles, and an indistinct median vitta on disk of pronotum; visible portion of costal margin of fore wing; a pair of distinct or indistinct spots at basal tumescence, and a narrow vitta along midline starting slightly posteriad of basal tumescence and fading away before posterior margin of scutellum; legs ochraceous, antennae slightly darker, brownish ochraceous; venter of head brown, ventrally visible surfaces of mandibular plates and clypeus ochraceous, labium ochraceous, thoracic pleura (except explanate lateral portion of propleuron) and abdomen dark brown, lateral margin and an adjoining sublateral spot at anterior margin of each of ventrites III–VII ochraceous. Integument and vestiture as in the generic description.

*Structure* mainly as in generic description. *Head* with lateral margin of mandibular plates distinctly emarginate anteriad of eye (male, female), then strongly broadened, anteriormost portion rounded. Pronotum with anterolateral angles strongly produced anteriad, distinctly surpassing anterior margin of eye in both sexes but more strongly in male.

*External male genitalia.* Genital capsule (Figs 14–15) broadly rounded laterally. Paramere as in Figs 22–23. Phallus generally similar to that of *Hemitrochostoma altilabris*, it was not examined in detail.

*Female terminalia* (Fig. 32). Posterior margin of ventrite VIII more broadly emarginate than in *Hemitrochostoma altilabris*, proximal margin of valvifer VIII rather rounded; laterotergite IX distinctly broader than that of *Hemitrochostoma altilabris*. The single available female was not dissected.

*Measurements* (in mm) (holotype male / paratype female). Body length 6.6 / 6.6; median length of head to apex of clypeus 1.58 / 1.57, to apex of mandibular plates 2.00 / 1.84, greatest width (across mandibular plates subapically) 1.82 / 1.40, width across eyes 1.78 / 1.76, interocular distance 1.31 / 1.30, interocellar distance 0.64 / 0.66; oculo-ocellar distance 0.33 / 0.31, lengths of antennal segments I: IIa: IIb: III: IV as 0.74 / 0.63: 0.12 / 0.11: 0.82 / 0.78: 0.86 / 0.76: 1.01 / 0.90; median length of pronotum 1.40 / 1.76, humeral width 3.50 / 3.72; median length of scutellum 3.10 / 3.75, greatest width 3.66 / 4.25.

#### Etymology.

The specific epithet of the new species is the Latin noun *rutabulum* meaning 'shovel or spatula' (for cooking), referring to the broad, spade-like head of both sexes but particularly the male. Noun in apposition, not to be declined.

#### Distribution.

The species is known only from the type locality in Perak, Peninsular Malaysia (Fig. 33).

#### Bionomics.

Unknown.

## Discussion

The systematic relationships of *Hemitrochostoma* are uncertain. Morphological similarities of *Hemitrochostoma* and *Labroplatys* Rédei & Bu, 2013 were discussed by Rédei and Bu (2013).

## Supplementary Material

XML Treatment for Hemitrochostoma

XML Treatment for Hemitrochostoma altilabris

XML Treatment for Hemitrochostoma rutabulum
